# Neuropsychological performances, quality of life, and psychological issues in pediatric onset multiple sclerosis: a narrative review

**DOI:** 10.1007/s10072-023-07281-y

**Published:** 2023-12-29

**Authors:** Samuela Tarantino, Martina Proietti Checchi, Laura Papetti, Gabriele Monte, Michela Ada Noris Ferilli, Massimiliano Valeriani

**Affiliations:** 1 Developmental Neurology Unit, Bambino Gesù Children’s Hospital, IRCCS, Rome, Italy; 2 Systems Medicine Department, University of Tor Vergata, Rome, Italy; 3 Center for Sensory-Motor Interaction, Aalborg University, Aalborg, Denmark

**Keywords:** Multiple sclerosis, Children, Adolescents, Cognitive performance, Psychiatric comorbidity, Quality of life

## Abstract

Multiple sclerosis (MS) is primarily a disease diagnosed in young and middle-aged adults. Although MS is a rare condition in pediatric age, an increasing rate of patients is diagnosed under the age of 18. The disabling nature of the disease cannot be reduced only to physical symptoms. Several additional symptoms such as cognitive impairment, fatigue, and psychological symptoms are common features of pediatric MS. The reviewed literature suggests that, despite the lower physical disability, children and adolescents diagnosed with MS are vulnerable to cognitive impairment even in the early stage of the disease. The neuropsychological profile of pediatric MS may resemble that of adult MS, including an impairment in attention/information processing speed, learning, verbal, and visuospatial memory. However, cognitive difficulties in children and adolescents are more likely to involve also general intelligence and linguistic abilities, presumably due to patients’ younger age and cognitive growth stage. Cognitive difficulties, beyond physical disability and relapses, may have a considerable impact on learning and school achievement. Depression and fatigue are other highly prevalent disturbances in pediatric MS and may contribute to patients’ low functional outcomes. Overall, these manifestations may cause considerable functional impairment on daily activities and quality of life that may require individualized rehabilitative treatment and extensive psychosocial care. Additional neuropsychological research evaluating larger samples, using more homogenous methods, and exploring the role of MS treatment on cognitive and psychological development is required.

## Introduction

Multiple sclerosis (MS) is a chronic, unpredictable, inflammatory disease that affects the central nervous system [1]. It is characterized by lesions in the brain and spinal cord that may lead to both physical and cognitive impairment [2]. Although MS is usually considered an adult disease (adult-onset multiple sclerosis (AOMS)), approximately 3–5% of all patients with MS experience their first demyelinating episode before the age of 18 (pediatric-onset multiple sclerosis (POMS)) [3, 4]. The incidence and prevalence of pediatric MS are not completely known. It has been estimated that the overall prevalence and incidence of POMS may range respectively from 0.7 to 26.9 and from 0.05 to 2.85 per 100,000 children a year [3]. The sex distribution (female/male) of POMS is relatively equivalent in prepubertal children, while in post-puberty, the distribution shows a more pronounced female dominance, similar to AOMS [3]. This marked female disparity with age suggests a possible connection between the onset of menstruation and disease expression.

Pediatric MS may show different clinical presentations according to age at onset. While younger children (< 11 years old) often show multifocal symptoms, patients aged > 12 years, as the adults, usually have a monosymptomatic onset [3]. The most common clinical presentations in childhood are optic neuritis (ON), transverse myelitis (TM), and acute demyelinating encephalomyelitis (ADEM) [5]. In particular, ADEM at onset occurs more often in children under 10 years [5]. At onset, children very often show brainstem symptoms (25–41%), sphincter dysfunction, motor problems (30%), and sensory disturbances (15–30%) [4]. Younger patients with MS are also more likely to show cerebellar dysfunction [6]. POMS has a highly inflammatory course and is clinically more active than AOMS [6]. Most children (over 90%) are diagnosed as having relapsing–remitting form and show a higher relapse rate compared to AOMS, especially during the first 2 years [3, 6]. Due to the greater plasticity in the developing nervous tissue and the higher myelin repair ability, pediatric patients have better relapse recovery with less neurologic impairment [7, 8]. Pediatric patients have a slower progression of disability [9]. However, they reach a significant disability earlier in adult life (as early as the third decade of life) than patients with AOMS [9, 10].

The disabling nature of the disease cannot be reduced only to physical symptoms. Several additional symptoms such as cognitive impairment, fatigue, and psychological symptoms are common features of MS [7]. Overall, these manifestations may cause considerable functional impairment on daily and social activities, academic achievement, and quality of life that may require individualized rehabilitative treatment and extensive psychosocial care [7, 11, 12]. Given the unpredictability of the disease course and its heterogeneous features, MS has a potential impact not only on patients’ cognitive and psychological development, but it may also lead to longstanding adaptive problems involving the entire family functioning.

Considering that childhood and adolescence are critical periods for appropriate educational attainment, social and personal growth, an analysis of the impact of the disease on patients is essential for an appropriate management.

### Aims

The aim of this narrative review article was to provide an extensive and up-to-date perspective of the impact of MS on affected children and adolescents. In particular, we investigated the current literature on neuropsychological impairment of children and adolescents who suffer from this disease, attempting to describe their long-term neuropsychological profile. In order to better understand patients’ global needs, we investigated the current literature data on common symptoms associated with MS such as fatigue, psychiatric comorbidity, and the impact of the disease on patients’ quality of life (QoL).

This review provides a comprehensive and current perspective on the impact of MS on children and adolescents affected by MS. Our paper contributes to the literature by offering an extensive and up-to-date exploration of the neuropsychological impact of the disease on children and adolescents. By delving into long-term effects, including fatigue, psychiatric comorbidities, and the impact of MS on quality of life, our paper enriches the understanding of pediatric MS. Beyond contributing to the current understanding of multiple sclerosis in this age cohort, this work establishes a robust framework for future studies and targeted interventions. Our results underscore the critical need for appropriate management and comprehensive care for POMS patients during these pivotal developmental stages, emphasizing the necessity of tailored interventions to address their cognitive and psychological challenges.

## Methods

**Fig. 1:**
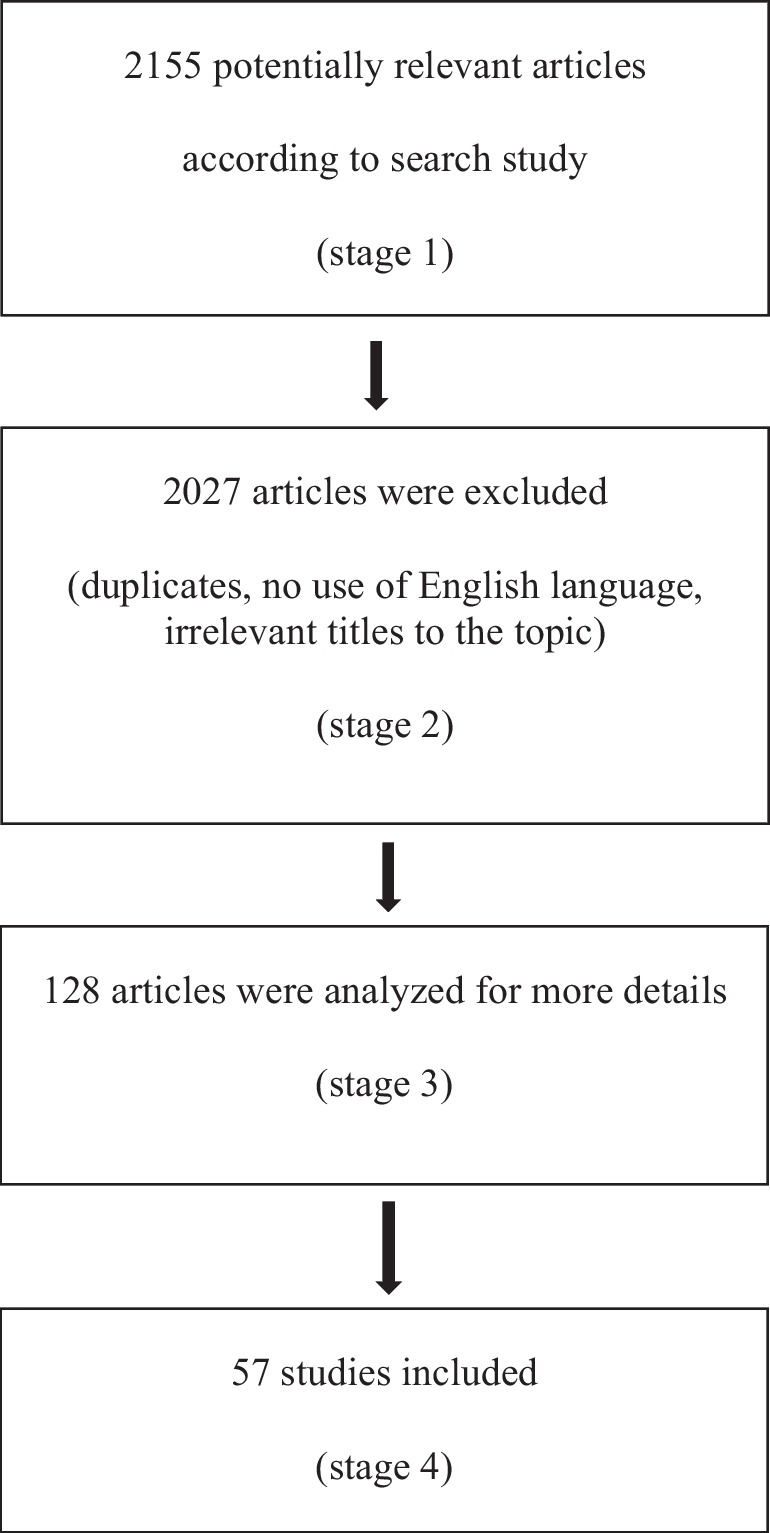
Flow diagram of the study methodology

In this narrative review, the research of appropriate papers was carried out using MEDLINE and Web of Science. Our research considered studies published up to February 2023. We considered only papers published in English language. Search terms included “Pediatric Multiple Sclerosis” or “Pediatric Onset Multiple Sclerosis” and “Cognitive impairment”, “Cognitive performance” or “Neuropsychology”, “Attention”, “Memory”, “Language”, “Processing speed” and “Intelligence”. Moreover, we included in our research “Social cognition”, “Psychiatric comorbidity”, “Fatigue”, and “Quality of life”. Our search included patients of an age ranging from 0 to 18 years. We considered also articles involving adult patients diagnosed before 18 years old. Observational, prospective, and retrospective studies were analyzed as well as clinical trials and multicentric studies (Fig. 1).

## Results

### Cognitive impairment

Cognitive impairment and neuropsychological dysfunctions are common and debilitating symptoms of MS [2]. It is assumed that cognitive dysfunction is not a late symptom of progressed MS, but it may be already detected even in the early stage of the disease [13, 14]. Cognitive dysfunctions have been found in all MS phases and may occur even in a pre-clinical stage or in the absence of major physical dysfunction [8, 15]. Moreover, research on both adult and pediatric age evidenced that low performance in selected cognitive domains may predict relapse and also disability progression [16].

Due to the onset of the disease during a crucial period for central nervous system maturation and development, pediatric patients with MS may be particularly vulnerable to cognitive impairment [7, 17, 18].

So far, there is lack of specific consensus on the assessment tools to be used, and the definition of cognitive impairment shows a large variability among several studies (Table 1) [7]. Despite this heterogeneity, research data consistently showed that approximately one third of patients under 18 years show cognitive impairment [7, 13, 16, 19]. The neuropsychological profile of pediatric MS may resemble that of adult MS, including an impairment in attention/information processing speed, learning, verbal, and visuospatial memory [7, 19]. However, cognitive difficulties in children and adolescents are more likely to involve also general intelligence and linguistic abilities, presumably due to patients’ younger age and cognitive growth stage [8, 17, 20–22].

**Table 1:** Longitudinal studies on cognitive performances

Author	Sample	Neuropsychological domains assessed (tests used)	Neuropsychological results and relationship with clinical features
Wallach et al. 2020	955 patients (almost two-thirds were female) 500 with POMS and 116 with CIS (a total of 383 underwent their follow-up evaluation) Mean age of symptom onset at initial evaluation, 13.5 years; IQR, 12.0–15.9 Follow-up length, 1.8 years (IQR, 1.0–2.7 years)	Processing speed (Symbol Digit Modalities Test)	- Baseline: 480/616 (77.9%) patients had intact or unimpaired cognitive processing speed - Follow-up: most patients (85.9%) did not show clinically meaningful change in cognitive processing speed In patients who experienced clinically decline, it was associated with older age of multiple MS onset and male gender
MacAllister et al. 2007	12 MS patients (8, F; 4, M) Mean age at disease onset, 12.5 ± 1.86 years Follow-up length, 21.58 ± 9.3 months; range, 11–30 months	- Attention, rapid visual scanning, and processing speed (Trail Making Test-A and B) - Verbal fluency (The Controlled Oral Word Association Test) - Confrontation naming ability (The Boston Naming Test) - Receptive language, verbal comprehension, and verbal memory (The Clinical Evaluation of Language Fundamentals-Listening to Paragraphs subtest) - Memory and Learning (Wide Range Assessment, Visual Learning subtest - Visual-motor integration skills (Beery-Buktenica Visual-Motor Integration test)	- Baseline: 10/12 patients (83.3%) demonstrated impaired performances on at least one neuropsychological task - Follow-up: 10/12 patients were impaired on one or more tests (patients were not the same patients in every instance) Low performance in attention/executive functions and memory Association between age of MS onset, baseline level of neurologic disability and cognitive decline
Portaccio et al. 2022	68 participants - 33 patients with MS (F, 17; M, 16) Mean age at MS onset, 11.7 ± 4 years - 35 healthy controls (F, 18; M, 17) Follow-up length, 12.8 ± 0.8 years from the baseline evaluation	- Verbal learning (RAO battery, Selective Reminding Test) - Visuospatial learning (RAO battery, Spatial Recall Test) - Speed processing (RAO battery, Symbol Digit Modalities Test) - Expressive language/executive functions (RAO battery, Word List Generation)	The initial worsening between the first evaluation and year 2, followed by partial improvement at year 5, was further compensated after 12 years However, 18/33 (54.5%) of patients fulfilled the criterion for cognitive impairment (more than double compared with baseline proportion of 21.2%) The most frequently impaired were the long-term verbal retrieval abilities Higher IQ (> 90) at baseline and lower number of relapses in the 2 years before baseline predicted better cognitive performances
Carotenuto et al. 2019	51 MS patients (F, 26; M, 25) - 33 patients (65%) had pediatric onset (< 18 years) - 18 patients (35%) had a disease onset between 18 and 25 years old Mean age at disease onset, 17.2 ± 3.9 years; range, 9–25 years Follow-up length, 5.52 ± 1.75 years; range, 2.6–8.3 years	- Verbal learning (RAO battery, Selective Reminding Test) - Visuospatial learning (RAO battery, Spatial Recall Test) - Speed processing (RAO battery, Symbol Digit Modalities Test) - Expressive language/executive functions (RAO battery, Tests Word List Generation)	Cognitive dysfunction was associated with an increased risk of relapses and disability progression In particular, low performance in attention/information processing and visual memory tasks were associated with increased physical disability and new relapses, respectively
Amato et al. 2014	94 participants - 48 patients with MS (F, 25; M, 23) Mean age at disease onset, 12.0 ± 3.8; range, 1.3–17.4 years - 46 healthy controls (F, 14; M, 32) Follow-up length, 4.7 ± 0.7 years from the baseline evaluation	- Verbal learning (RAO battery, Selective Reminding Test) - Visuospatial learning (RAO battery, Spatial Recall Test) - Complex attention (RAO battery, Symbol Digit Modalities Test; Trail Making Test-A and B) - Planning (Tower of London) - Expressive language (Aachener Aphasie Test, Semantic and Phonemic Verbal Fluency Test, and an Oral Denomination test subtests)	At 5 years, 18/48 (38%) fulfilled the criterion for cognitive impairment Comparing the 2-year and 5-year follow-up, 22.9% deteriorated, 10.4% remained stable, and 66.7% of the patients improved The most frequently impaired were verbal and spatial memory abilities. Tasks of attention/information processing speed and expressive language tended to improve A negative prognostic role of male, sex, younger age, and age at disease onset emerged (none of these variables was retained in the multivariate analysis)
Amato et al. 2010	106 participants - 56 patients with MS (F, 28; M, 28) Mean age at disease onset, 11.7 ± 3.8 years; range, 1.3–17.4 years - 50 healthy controls (F, 22; M, 28) Follow-up length, 2.1 ± 0.4 years from baseline evaluation	- Verbal learning (RAO battery, Selective Reminding Test) - Visuospatial learning (RAO battery, Spatial Recall Test) - Complex attention (RAO battery, Symbol Digit Modalities Test; Trail Making Test-A and B) - Planning (Tower of London) - Expressive language (Semantic and phonemic verbal fluency test; Aachener Aphasie Test, Oral Denomination subtest)	At 2 years, 39/56 patients (70%) fulfilled criterion for cognitive impairment Complex attention, verbal memory, verbal fluency, and receptive language were the most impaired skills The only significant predictor of cognitive deterioration was older age of the subjects
MacAllister et al. 2005	37 MS patients (8 patients underwent their follow-up evaluation) Mean/median age at symptom onset, 13.51/14 ± 2.56 years; range, 4–17 years Follow-up length, 1 year	- General intelligence abilities (WISC-III) - Attentional functions (Trail Making Test-A and B) - Verbal fluency (The Controlled Oral Word Association Test) - Confrontation naming ability (The Boston Naming Test) - Receptive language, verbal comprehension, and verbal memory (The Clinical Evaluation of Language Fundamentals, Listening to Paragraphs subtest) - Memory and Learning (Wide Range Assessment, Visual Learning subtest - Visual-motor integration skills (Beery-Buktenica Visual-Motor Integration test)	- Baseline: 13/37 (35.1%) were cognitively impaired - Follow up: 3/8 who were cognitively impaired at baseline went on to further decline Memory and attention were among the most common impaired skills Correlation between neuropsychological performance and the total disease duration, disability and number of relapses
Krupp et al. 2023	237 participants - 72 with POMS (F, 49; M, 23) - 99 pediatric healthy controls (F, 63; M, 36) - 66 adults with AOMS (F, 46; M, 19)	- Information processing speed (Symbol Digit Modalities Test of the BICAMS Battery and CogState Brief Batteries) - Verbal memory and visuospatial (Rey Auditory Verbal Learning Test; BICAMS battery, Brief Visuospatial Memory Test-Revised subtest)	No difference was found between pediatric MS and healthy control groups Pediatric patients had better performance than adults with MS
Marin et al. 2013	4 MS patients (F, 2; M, 2) Mean age at disease onset, 7.4 years; range, 5.3–10.8 years Follow-up length, 11.9 years	- General intelligence abilities (WISC-III) - Attention, rapid visual scanning, and processing speed (Trail Making Test-A and B) - Visual-motor integration skills (Beery Visual-Motor Integration Test) - Manual Dexterity and Visual-Motor Coordination (Grooved Pegboard) - Lexical retrieval (Controlled Oral Word Association Test)	Most pronounced decline was found in processing speed (visuomotor speed), mental set shifting, and sequencing Global intellectual ability and phonemic fluency remained stable or improved over time No association between disease duration and relapses was found
Akbar et al. 2017	82 MS patients (F, 51; M, 31) Mean age at disease onset, 11 ± 3.6 years Follow-up length, up to 4 years	- General intelligence abilities (WISC-R; WASI) - Verbal learning (RAO battery, Selective Reminding Test; Test of Memory and Learning-II, Selective Reminding) - Visuospatial learning (RAO battery, Spatial Recall Test) - Speed processing (RAO battery, Symbol Digit Modalities Test) - Planning (Tower of London) - Attention/executive functions (Trail Making Test-A and B; Conners’ Continuous Performance Test; Wisconsin Card Sorting Task; Delis Kaplan Executive Function System, Phoonemic Verbal Fluency subtest) - Expressive language (Aachener Aphasie Test; Semantic and Phonemic Verbal Fluency Test; Woodcock Johnson-Revised Tests, Rapid Picture naming and Pictured vocabulary subtest) - Visual-motor integration skills (Beery-Buktenica Visual-Motor Integration test)	Processing speed increased with age, followed by a subsequent decline Older age at disease onset and higher IQ was associated with greater gains of cognitive functions
Banwell et al. 2005	10 MS patients: - 3 with recent first MS attack (within 12 months of testing) Mean age at disease onset, 8.4 years - 7 with remote first MS attack (mean time since first attack 5.1 years, range 2.2–7.9 years) Mean age at disease onset, 13.8 years	- Language (Clinical Evaluation of Language Fundamentals; Controlled Oral Word Association Test; Test of Language Competence) - Visual-motor integration skills (Beery-Buktenica Visual-Motor Integration test) - Memory (Rey-Osterreith Complex Figure; Children’s Memory Scale) -Learning (Children’s Auditory Verbal Learning Test; Woodcock Johnson-Revised Tests of Achievement) - Academic skills assessment (Wide Range Achievement Test)	Cognitive impairment, on at least one test, in all 10 children Deficits were almost exclusively in children with a remote disease onset Deficits mostly involved executive functions, processing speed or working memory
Öztürk et al. 2020	55 MS patients (F, 44; M, 11) and 53 health control sample of similar age (46 patients completed their follow-up evaluation) Mean age at symptom onset, 13 ± 2.6 years; range, 6–16 years Follow-up length, 2 years	- Non-verbal intelligence abilities (Progressive Matrices of Raven) - Attention and interference in reaction time (The Stroop Effect) - Visuospatial judgement (The Benton Judgment of Line Orientation Test) - Verbal fluency (KAS-Animal Test)	- Baseline: 16/46 (34.7%) patients failed in at least 3 of 4 tests - Follow-up: 22/46 (47.8%) patients failed in at least 3 of 4 tests The most affected domains were non-verbal reasoning and attention/concentration Patients with disease onset before age 12 years showed an impairment in non-verbal reasoning. Other functions improved over time in patients and control groups
Till et al. 2013	28 MS patients (F, 22; M, 6) and 26 age-matched controls Mean age at disease onset, 11.6 ± 3.8 years Follow-up length, 1-year period	- General intelligence (WASI) - Attention and processing speed (Tests of Cognitive Abilities, Visual Matching; Trail Making Test-A and B; the Symbol Digits Modalities Test; the Conner’s Continuous Performance Test) - Language (WASI, Similarities subtest; Woodcock-Johnson Tests of Cognitive Abilities, the Picture Vocabulary test; Delis-Kaplan Executive Function System, Verbal Fluency subtest) - Visuospatial ability (Beery Visual-Motor Integration Test; WASI, Block Design and Matrix Reasoning subtests) - Memory (Test of Memory and Learning–2nd edition, Word Selective Reminding, Memory for Stories, Abstract Visual Memory, and Facial Memory subtests) - Academic achievement (Woodcock-Johnson Tests of Cognitive Abilities, Calculation, Spelling, Letter-Word Identification, and Passage Comprehension subtests) - Fine motor dexterity (Grooved Pegboard test)	Only 4/22 (18%) patients compared to 19 (86%) of control group demonstrate age-expected improvements in cognitive performance Patients showed decline mainly on tests of attention and processing speed, abstract visual memory, visuomotor integration, calculation, verbal fluency and spelling Longer disease duration associated with worsened visuomotor integration
Charvet et al. 2014	67 patients (F, 42; M, 25) 62 with POMS and 5 with CIS Mean age of symptom onset at initial evaluation, 12.94 ± 3.13 years; range, 3.15–16.81 years Follow-up length, 1.64 ± 0.63 years; range, 0.75–3.38 years	- General intelligence abilities (WASI, WASI-II) - Total trial learning and long-term delayed free recall (California Verbal Learning Test) -Receptive and expressive vocabulary skills (Expressive One-Word Picture Vocabulary Test) -Speed and accuracy of decoding skills (Wechsler Individual Achievement Test-Pseudoword Decoding subtest) - Visual-motor integration skills (Beery-Buktenica Visual-Motor Integration test) - Memory: from the (WISC-IV, WAIS-IV- Digit Span subtest) - Visual scanning, number sequencing, letter sequencing, number/letter switching, and motor speed (Delis-Kaplan Executive Functioning System- Trail Making Test)	- Baseline: 37.3% of patients showed cognitive impairment - Follow-up: 32.3% of patients evidenced cognitive impairment Attention, speeded processing, and visuomotor integration were the most commonly impaired skills Decline at follow-up was not associated with any clinical features

*POMS* pediatric onset multiple sclerosis, *CIS* clinically isolated syndrome, *IQR* interquartile range, *MS* multiple sclerosis, *RAO* Rao’s Brief Repeatable Battery of Neuropsychological Tests, *WISC-III* Wechsler Intelligence Scale for Children—Third edition, *AOMS* adult onset multiple sclerosis, *BICAMS* Brief International Cognitive Assessment for Multiple Sclerosis, *WISC-R* Wechsler Intelligence Scale for Children-Revised, *WASI* Wechsler Abbreviated Scale of Intelligence, *WASI-II* Wechsler Abbreviated Scale of Intelligence—Second edition, *WISC-IV* Wechsler Intelligence Scale for Children—Fourth edition, *WAIS-IV* Wechsler Intelligence Scale—Fourth edition

In an early study [23], Kalb et al. described low intelligence performance, weakened verbal fluency, and perceptual motor difficulties among a small sample of patients with MS (*n* = 9). This early study, however, did not explore other cognitive functions commonly affected in adults, such as memory and attention. Several years later, MacAllister et al. examined the neuropsychological profile of 37 pediatric patients with MS [22]. Cognitive impairment, defined as a performance falling 1.5 or more standard deviation below normative range on at least two cognitive tasks, was found in one third of patients (35.1%). Moreover, 59% of patients had a low performance on at least one neuropsychological test. The most impaired domains were complex attention (e.g., rapidly shifting attention between competing stimuli) (29.7%) and delayed recall of verbal and visual information (respectively, 18.9% and 11%). Language abilities were frequently affected, with low performances in naming (18.9%) and receptive language (13.5%) [22]. The study described an association between cognitive functioning and the total disease duration, total number of relapses and disability (measured by Expanded Disability Status Scale, EDSS).

These studies, however, were limited by the lack of a matched control group. In a multicenter study, Amato et al. compared the neuropsychological functioning of pediatric patients diagnosed with MS (*n* = 63) with that of a control healthy group (*n* = 57), confirming the vulnerability to cognitive impairment in MS group [24]. The neuropsychological evaluation showed significant cognitive impairment (defined by scores falling below the 5th percentile of healthy control on at least three tests) in 19 patients (31%), whereas 32 patients (53%) showed a failure at least in two tests. As in other studies, prominent weakness was found in memory, attention, information processing speed, and executive functions. Exploring the global intelligence profile, the authors found lower scores in MS group in both verbal and performance IQ. In particular, the neuropsychological profile was characterized by low performance in verbal comprehension and, in a lower degree, semantic and phonemic fluency, thus confirming the peculiar impairment in language [24]. Among the possible variables associated with the low cognitive skills in these patients, the only significant predictor was the IQ score in the inferior range (lower than 90) which, in turn, was related to younger age at onset [24]. These findings were confirmed by Till et al., who described a reduction of attention and processing speed (38%), visuomotor integration (23.5%), and expressive vocabulary abilities in MS group compared with control group [25]. Moreover, the study showed an association between higher IQ and older age at disease onset, shorter MS duration and lower EDSS. Using a computerized neurocognitive battery, a more recent study demonstrated reduced accuracy on tests of attention/inhibition, visuospatial processing working memory and verbal memory (after adjusting for response time), in adolescents and young adults with POMS, compared with healthy control [26]. On the other hand, the absence of significant differences between cognitive performances among pediatric patients compared with a control healthy group was described in a recent study by Krupp et al. [27]. When compared with a group of adult patients with MS, the pediatric sample showed a better performance. A higher repair ability and better relapse recovery have been suggested as causal mechanisms.

However, beyond the age-conferred resilience, differences in the study groups could explain the discrepancies in comparison to older studies [28, 29]. In particular, patients in Krupp et al. study had shorter disease duration and were almost all (93%) treated with disease modifying therapies (50% using high efficacy drugs). These results support the role of DMT in improving the disability progression outcomes in individuals recently diagnosed [30].

Results on the influence of clinical factors on cognitive impairment are inconsistent and far from being conclusive [7]. Although a consistent body of research in POMS described lower cognitive performance in patients with neurological disability [13, 14, 25], other studies have shown a weak relationship between cognitive dysfunctions and the level of disability, indicating that low cognitive skills may occur independently from neurological disability [7, 11, 21]. Some authors supposed that cognitive functioning may be considered an early marker of disease activity and a prognostic factor for disease progression. Interestingly, cognitive decline can even predict the presence of structural abnormalities in the magnetic resonance imaging (MRI) [31].

The role of age of onset and disease duration on cognitive decline is still debated. In recent years, an increasing body of research examined the long-term evolution of cognitive functioning in pediatric MS, but the results are still inconclusive, showing heterogeneous outcomes over the time [7, 32–34]. A growing body of research showed higher risk of cognitive impairment in adults with POMS [28, 35].

Banwell et al. compared a small group of children with recent (disease onset within 12 months of testing; *n* = 3) and remote first MS attack (*n* = 7) [36]. The study demonstrated not only a cognitive decline on at least one test in all patients (*n* = 10) but also a higher risk of neuropsychological impairment in children with longer disease duration and younger age at onset. These findings were in accordance with two later studies of MacAllister et al. who reported a worsening of cognitive performance, in particular attention and memory, even in a relatively short time interval [14, 22]. Moreover, the authors highlighted the role of the disease duration, disability, and total number of relapses on cognitive disturbances [22]. In a 2-year follow-up study by Öztürk et al. [37], the authors observed a high percentage of cognitive impairment in pediatric patients with MS (47.8%), with significant differences with a control healthy group. The most affected skills were non-verbal reasoning and attention/concentration. Exploring the main clinical factors associated with the cognitive performance, the authors found that age at disease onset and EDSS appeared as important factors predicting cognitive functions. A 15-month follow-up by Till et al. showed that, although most patients had a relatively stable cognitive performance, healthy controls were more likely to have improvement in multiple cognitive domains [38]. A deterioration in cognitive skills, defined as significant decline on three or more tests, was found in 25% of patients as compared to only 3.8% of controls. The most commonly impaired skills were spelling, verbal fluency, visual memory, processing speed, and calculation. Longer disease duration was associated with greater deterioration in visuomotor integration. These findings led the authors to suppose that the cognitive decline of pediatric MS patients could be attributable to a lack expected maturational trajectory compared with healthy peers. While cognitive deterioration appears to be relatively frequent in pediatric MS, some studies evidenced little changes or even an improvement in cognitive skills over the time. In a follow-up of 1.8 months involving 383 patients with POMS or clinically isolated syndrome (CIS), Wallach et al. found that most patients (85.9%) did not show clinically meaningful change in processing speed [13]. Impaired processing speed occurred in 14.1% of POMS at the second assessment (14.0% of CIS). Factors associated with clinically neuropsychological decline included older age at MS onset and male gender. A low incidence of cognitive decline was also described by Charvet et al. [39], who found a relatively stable impairment from baseline (37% of patients) to follow-up (33%) (mean interval of 1.64 years) in a pediatric population of 62 MS and 5 CIS patients.

Heterogeneous results emerged over time in a 12-year-long follow-up by Portaccio et al. [15]. In a cohort of patients with MS assessed at 4 time points (baseline, 2, 5, and 12 years), a global worsening of cognitive performance was noticed at year 2, while there was an improvement at years 5 and 12 (with no significant variation between them). The most impaired domain was the verbal learning, with 41.9% of the sample showing difficulties in these skills. In disagreement to other studies, both disease duration and age of onset did not impact neuropsychological functioning. Higher clinical disease activity before the baseline evaluation was predictive of cognitive worsening. The study described the role of cognitive reserve against neuropsychological deterioration. Indeed, having an IQ ≥ 90 at baseline and a lower number of relapses in 2 years before the baseline were associated with better cognitive performances.

### Social cognition

Social cognition refers to a complex set of mental processes necessary to perceive, process, and interpreter social stimuli and the environment [40]. One essential domain of social cognition is the Theory of Mind (ToM), which is defined as the ability to understand others’ mental state and ascribe intents, desires, and beliefs of others [40]. Over the last decades, a growing body of research explored social cognition in several neurodegenerative conditions, evidencing defective social cognition, in particular emotional processing and ToM [40]. Data investigating social cognition in MS patients are sparse [40, 41]. To the best of our knowledge, only three studies included patients with POMS. In a pilot study, Charvet et al. evidenced that social cognition may be an area of cognitive functioning affected in POMS population [40]. Comparing a group of 28 patients with POMS with 32 healthy controls, the authors found worse scores on ToM tasks in patients. In particular, lower performance in both cognitive and affective domains of ToM was found. Results on the affective task were similar to those emerged in other clinical disorders (i.e., psychiatric impairment). The impairment in ToM was not influenced by low cognitive abilities suggesting that, in pediatric MS, these deficits may be independent. The clinical factors showing a weak to moderate negative correlation with ToM were the total number of relapses and a longer history of disease. In 2018, Neuhaus et al. confirmed low social cognition abilities in pediatric patients with MS, but no correlation with both disease duration and EDSS was found [41]. Moreover, these authors described a particular impairment of complex social situations and facial affect recognition. More recently, Massano et al. compared classical and social cognition in 30 patients with POMS with two groups of patients with AOMS: the first matched for disease duration, and the second matched for age [42]. While the study confirmed the frequent cognitive impairment in POMS, no difference emerged in ToM skills between patients with POMS and AOMS. However, analyzing ToM abilities according to the age of MS onset, the authors found significant lower performances in patients with age of disease onset ≤ 15 years.

### Psychiatric comorbidity

Psychiatric comorbidity in patients with MS was initially noticed by Charcot, who described hallucinations, depression, and mania among other manifestations of the disease [43]. Despite a large body of research on adults with MS, data on pediatric age are so far limited. However, an increasing number of studies suggest that psychiatric disorders may be common also in pediatric MS [7, 15, 19, 20, 44, 45]. In an early study by MacAllister et al. on pediatric patients with MS, nearly half of the patients who underwent a psychiatric evaluation received a formal diagnosis, most of them suffering from anxiety and/or depression [22]. The high prevalence of anxiety and mood disorders in pediatric patients with MS was confirmed in later studies. In 2010, Goretti et al. observed that about 30% of pediatric MS cases fulfilled criteria for a formal diagnosis of affective disorders which included anxiety, panic, major depression, and bipolar disorders [45]. On the other hand, on a self-report questionnaire exploring depression symptoms, only 17% of the patients had scores in the clinical range. The authors supposed that self-report inventories could be not sufficiently sensitive to detect subclinical psychiatric symptoms and highlighted the importance of structured interviews for a more precise evaluation of affective disorders in children and adolescents. Aiming at investigating the prevalence and risk factors associated with emotional and behavioral outcomes in adolescents with MS, Till et al. evaluated a sample of 31 pediatric patients with MS and 31 healthy subjects [12]. Compared with controls, patients more frequently showed problems of lower self-reliance and inattention/hyperactivity. Although a significant difference was not found, the adolescents with MS showed also elevated symptoms of anxiety (31%), depression, and somatization (24.1%). Similar results emerged from the parents, who described elevated symptoms of depression (29%), but also difficulties in adaptive behavior and somatization. The study also examined how emotional and behavioral functioning related with clinical factors. Psychosocial outcomes were not associated with brain lesion volume, IQ, disease duration, or number of relapses. On the other hand, functional status (evaluated by the EDSS) positively correlated with parent-reported internalizing symptoms in their children. Also, later studies showed the tendency of parents to describe higher rate of mood disorders in their children with MS as compared with patients’ self-report. In a study by Charvet et al., 140 pediatric patients with MS or CIS were evaluated with BASC-2 (Behavior Assessment System for Children-2). The study evidenced that all BASC-2 scales were in the typical range in both self and parents’ reports [46]. However, about 33% of the sample reported a clinically significant problem on at least one scale. Although mood and behavioral problems were rated by patients as slightly lower than parents’ reports, both indicated anxiety, somatization, and attention problems as the most common disorders. While no association between these problems and disease features or fatigue was found, the authors described a higher rate of emotional and behavioral problems in patients with cognitive impairment. These results agree with a previous study on 45 pediatric MS patients, where a higher rate of cognitive impairment was found in patients diagnosed with anxiety or mood disorder [47].

Very few studies explored the comorbidity between pediatric MS and psychiatric disorders, such as psychosis, autism, or ADHD. In a retrospective study, Pakpoor provided the first evidence of an association between childhood MS (and other demyelinating diseases) with psychotic disorders [48]. The study evidenced that while psychotic disorders did not significantly precede the demyelinating disease, autism and ADHD were associated with it. These results may reflect the different typical age in which psychosis, ADHD, and autism are diagnosed. One year later, in a nationwide population-based study, Boesen explored the presence of psychiatric comorbidity before and after onset of MS [49]. A higher rate of psychiatric comorbidity was described among patients with MS, especially girls, compared with children without MS. While an increasing rate of anxiety and depression was found compared with the pre-MS age, no significant higher risk for MS was found among children exposed to psychiatric morbidity during the last 2 years. According to these findings, psychiatric comorbidity may start after MS onset, although undiagnosed cognitive/psychiatric problems preceding the illness onset cannot be excluded.

### Fatigue

Fatigue related to MS is defined as “a subjective lack of physical and/or mental energy that is perceived by the individual or caregiver to interfere with usual or desired activities” [50]. The MS International Federation recognized fatigue as either physical or cognitive [51]. With an estimated prevalence ranging from 9 to 76%, fatigue is a common, pervasive, and disabling symptom of MS [52]. Unfortunately, since there are few validated questionnaires for children/adolescents’ fatigue, this symptom is often assessed by multi-rater perspectives which may lead to discrepant results. Indeed, parents are more prone to report higher fatigue than children or adolescents [18, 34, 52, 53]. This disagreement may be influenced by parents’ moods or excessive parental worry [52–54].

Although both fatigue and cognitive problems may have a negative impact on quality of life, data on their relationship in pediatric patients with MS are sparse and far from conclusive.

Comparing the levels of fatigue in pediatric patients with MS, chronic fatigue syndrome (CFS), and healthy subjects, Carrol et al. reported similar levels in both patient groups [55]. Cognitive difficulties emerged in both non-fatigued and fatigued children with MS, suggesting that neuropsychological impairment is independent of fatigue, as confirmed by a more recent study [15].

While fatigue is not related to the overall cognitive functioning, it can correlate with the scores obtained in individual neuropsychological tasks [34]. Goretti et al. found an association between cognitive fatigue and performance in individual neuropsychological tasks [53]. Higher scores of self-reported cognitive fatigue were associated with low performance in a problem-solving task, whereas higher levels of parent-reported cognitive fatigue were associated with low scores in tests of processing speed, complex attention verbal comprehension, and verbal learning.

Consistent evidence supports the reciprocal relationship between fatigue and depressed mood in pediatric MS. Several studies described a moderate to strong association between fatigue and depression [12, 52–54, 56]. Also a moderate association between fatigue and self-reported anxiety has been reported [57].

As in patients with AOMS, also in those with POMS, fatigue is independent of age at MS onset, disease duration, relapse rate, and number of relapses [52, 54, 56].

### Quality of life

MS may impair participation of children and adolescents in curricular and social activities, leading to a low QoL. In an earlier study, Boyd and MacMillan identified developmental, psychological, and social experiences adversely affected by the diagnosis of MS [58]. The most common stressors were unpredictable relapses, unresolved symptoms, uncertainty about the future, demanding treatment regime, changes in peer relationships, and family conflicts (Table 2).

**Table 2:** Main results of studies on quality of life

Author	Sample	Neuropsychological domains assessed (tests used)	Results on quality of life and factors associated
Lanzillo et al. 2016	54 MS/CIS patients (F, 31; M, 23) - Pediatric group (*n* = 34): disease onset < 18 years - Juvenile group (*n* = 20): disease onset 18–25 years	PedsQL	QoL was higher in pediatric onset than juvenile onset MS patients Low QoL showed an association with disability, age of disease onset, and depression
Holland et al. 2014	26 patients (F, 17; M, 9) 23 SM and 3 CIS	PedsQL (emotional scale)	Both self- and parent-report reported severe difficulties in around 30% of participants An association between emotional difficulties, fatigue, and executive dysfunction was found
MacAllister et al. 2009	51 MS patients (F, 33; M, 18) Mean age at disease onset, 13.1 ± 2.80 years; range, 3–16 years	PedsQL	Social domain was the less affected QoL domain - 20% of patients reported severe difficulties in physical scale - 10% in emotional scale - 4% in social scale - 28% in school scale A significant association between disability, fatigue, and quality of life variables was found
Storm van’s Gravesande et al. 2019	316 participants - 106 MS patients (F, 76; M, 30) Mean age at onset, 14.1 ± 2.4 years; range, 4–18 years - 210 healthy subjects (F, 95; M, 115)	PedsQL	Self-reported QoL was lower in all domains in MS patients compared to control except for the Social Functioning Scale Loss of QoL was predicted by disability, fatigue, and depression
Florea et al. 2020	37 patients (F, 23; M, 14) - 26 MS patients (F, 9; M, 17) Mean age at MS onset, 12.4 ± 3.1 years	PedsQL	School and emotional domains were compromised in about half of the children and parents reported - 40% or patients reported poor QoL in total score - 20% in physical scale - 50% in emotional scale - 5% in social scale - 50% in school scale Parents reports tended more difficulties in QoL than patients reports
Schwartzal et al. 2018	66 patients (F, 44; F, 22) Mean age at disease onset, 13.20 ± 3.91 years	PedsQL	Both self and parent reports were in the normal range with the exception of parent-reported school functioning
Mowry et al. 2010	- 50 MS/CIS patients (F, 31; M, 19) -12 control group (sibling of patients; F, 9; M, 4) - 38 children with neuromuscular disorders (F, 17; M, 34)	PedsQL	Patients with MS or CIS had reduction in almost all aspects of QoL compared with their siblings Compared with children with neuromuscular disease, MS patients reported better total QoL Children with MS or CS tended to have worse self-reported school functioning and emotional, while those with neuromuscular disorders reported substantially worse social functioning The main predictor of reduced QoL was greater neurologic disability
O’Mahony et al. 2019	236 patients - 58 MS patients (F, 39; M, 19) - 178 monoADS patients (F, 81; M, 97)	PedsQL	No differences between self-report QoL of children with MS and those with monoADS A reduced QoL and emotional functioning in MS compared to those with monoADS emerged in parent reports Diagnosis of MS was negatively associated with parental QoL and family functioning
Rosa et al. 2022	17 SM patients (F, 11; M, 6) Mean age at disease onset, 17 ± 2.9 years; range, 12–35 years Follow-up length, 4 years	PedsQL	An increase in the social functioning subscale and a decrease in the emotional subscale. No longitudinal changes were observed in other domains The change in social functioning was affected by the occurrence of relapses
Marrie et al. 2020	122 participants - 36 MS patients (F, 27; M, 9) Mean age at symptom onset, 13.62 ± 3.14 Follow-up length, 2.60 ± 2.04 - 43 monoADS patients (F, 19; M, 24) Mean age at symptom onset, 6.49 ± 3.82 Follow-up length, 3.60 ± 1.54 - 43 healthy controls (F, 28; M, 15)	PedsQL	QoL did not differ between groups; however, physical domain was lower in children with MS than in the other groups Children with MS with neurologic abnormalities reported lower physical QoL No association between odds of hospitalization and QoL
Toussaint-Duysteret al. 2018	38 patients (F, 25; M, 13) - 22 MS patients (F, 18; M, 4) Median age first symptoms, 14 years; IQR, 9.0–17.0 years - 16 ADEM patients (F, 7; M, 9) Median age first symptoms, 4.5 years; IQR, 1.5–11.5 years	PedsQL	Except emotional scale, patients reported low QoL scores. Parent reports showed low score in all domains Fatigue, but not the other parameters, was significantly correlated with QoL
Lulu et al. 2014	30 MS patients (F, 16; M, 14)	PedsQL	Median scores tended to be lower in psychosocial aspects Disability was associated with a lower score on quality of life. Higher cognitive scores were associated with higher QOL (not when adjusting for patients’ age)
Ostojic et al. 2016	21 MS patients (F, 15; M, 6) Mean age at disease onset, 13.98 ± 2.29 years; range, 8–17.50 years	KIDSCREEN-52	In adolescents with MS physical self-report QoL domain were most likely to be compromised Functioning in other domains are relatively preserved Severity of the disease, duration, fatigue, anxiety, and depressive symptoms, was significant correlates of QoL

*MS* multiple sclerosis, *CIS* clinically isolated syndrome, *PedsQL* pediatric quality of life inventory, *QoL* quality of life, *monoADS* monophasic acquired demyelinating syndromes, *ADEM* acute disseminated encephalomyelitis, *IQR* interquartile range

Children and adolescents with MS may have worse QoL in all areas of life, compared not only with healthy subjects but also with children suffering from other neurological diseases [59]. The disease most frequently affects school and emotional domains, while a smaller impact on the social and physical areas has been reported [54, 59–64]. MS patients show worse scores in the emotional and school areas, compared to children with neuromuscular diseases [59, 62]. In pediatric patients with MS, some studies reported a high number of absences from school [21–23, 59]. They can be associated with sick days, relapses, hospital admissions, medical appointments, and therapy side effects [3, 6, 19, 59, 65]. However, other factors, such as disease course, cognitive impairment, and fatigue, may explain the changes in school QoL [13, 17, 22, 54, 56, 59, 66]. The importance of cognitive impairment in this contest is suggested by the association between higher cognitive scores and higher total QoL score [67]. Also, fatigue may contribute to a poor school QoL [56, 59]. As described by Carroll, tiredness in children and adolescents with MS reduces cognitive functions and causes daytime sleepiness, thus worsening their performance at school [55]. Parrish et al. showed that most patients with pediatric MS require special education plans or classroom accommodations (e.g., reduced workload or extended time on exams) [19]. Compared with the general population, patients with POMS show higher level of school dropout, lower educational achievement, and lower salaries [7, 15]. Beyond cognitive impairment and fatigue, psychiatric comorbidity may have an adverse influence on quality of life in children and adolescents with MS [11, 56, 58, 68]. Patients with MS suffering from depression have significant lower QoL, compared to healthy subjects [56, 68].

Studies on social sphere of QoL did not provide homogenous results. According to Goretti et al., impairment in social relationships may be found in 28% of patients, particularly due to tendency to isolation and behavioral changes, such as aggressiveness [45]. On the other hand, in a study by Storm Van’s Gravesande [56], the social functioning dimension was the least affected. In a more recent paper, Rosa et al. described increased social functioning over a 4-year follow-up [64].

Several studies showed that physical dimension of QoL is altered in pediatric patients with MS [54, 56, 59, 67]. In pediatric age, every type of physical impairment or change, though with mild disability, may lead to lower QoL [56]. However, the low levels of disability, typical of children and adolescents with MS, may explain the low impact of the disease on physical QoL [59, 60, 62, 64].

#### Disease modifying therapies, cognitive, and quality of life outcomes

Studies investigating the impact of pharmacologic interventions on cognitive outcomes in children and adolescents affected by MS are limited [11, 21, 69]. However, most patients included in studies on neuropsychological outcome of POMS were receiving disease modifying therapies (DMT) [8, 15, 17, 37]. In the study by Portaccio et al., the percentage of patients treated with highly effective DMT was higher in cognitively preserved patients (60% vs 38.9%), although the difference was not significant [15]. While no significant effect of the type of DMT on processing speed has been found by Wallach et al. [13], the protective role of natalizumab and fingolimod on progressive cognitive decline has been described [70, 71]. In a 24-month follow-up of pediatric patients treated with natalizumab, an improvement of EDSS relapses rate and MRI measures, but also an increase of cognitive functions (evaluate by SDMT) was described by Margoni et al. [70]. In a 2.5-year follow-up by Johnen et al., cognitive performances were preserved or even ameliorated in patients who escalated to highly effective drugs (natalizumab or fingolimod); on the other hand, a deterioration of cognitive performance was observed in patients who remained in first-line platform therapy (β-interferon or glatiramer acetate) [71].

In addition to the effects of DMT on cognitive performance, there is a growing scientific interest in the effects of immunomodulatory therapies on psychosocial outcomes. Ghezzi et al. evaluated the QoL changes in adolescents receiving interferon-β1 administered using electronic autoinjection [72]. The authors showed potential long-term benefits and increased quality of life over time in both self (except for emotional sphere) and parent reports. In particular, school functioning showed the greatest increase in patients’ and parents’ self-reports. In another study by Krupp et al., fingolimod demonstrated greater efficacy than interferon-β1 in improving QoL [73]. These results led the authors to suggest that treatments demonstrating substantial efficacy in reducing relapse rates and favorable tolerability also have beneficial effects on the QoL of patients with POMS.

## Discussion

Pediatric MS is a progressive disease with an unpredictable course and a negative impact on patients’ physical, cognitive, and psychological well-being, with a significant consequence on family life as well [1, 7, 19, 20, 44, 63]. Children and adolescents with MS, in addition to facing physiological developmental tasks, may undergo cognitive sequelae, neurological symptoms, and treatment regimens. Childhood and adolescence are critical periods for appropriate academic achievements, personal and social growth [74]. All these factors suggest that the impact of the disease in developmental age may be more severe than in adulthood independently of physical disability [15, 17, 21].

The reviewed literature suggests that, despite the lower physical disability, children and adolescents diagnosed with MS are vulnerable to cognitive impairment even in the early stage of the disease [7, 13, 14]. It has been hypothesized that, in pediatric age, cognitive decline may be considered a sensitive measure of MS severity [16]. Even the failure to reach the age-expected cognitive maturation has been considered a sign of disease progression [18].

Cognitive difficulties, beyond physical disability and relapses, may have a considerable impact on learning and school achievement [8, 19, 22, 67].

The mechanisms underlining the high prevalence of cognitive impairment in pediatric MS are not fully understood. Pediatric age is characterized by brain growth, neural network maturation, and ongoing myelination in the central nervous system [20, 74]. Since in children the neuropathological process of MS occurs in a critical period, it is not surprising that cognitive capabilities, in particular language, are particularly vulnerable [19]. While the possible role of age of onset, length of the disease, and disability as vulnerability factors of cognitive decline is controversial, the importance of brain plasticity, cognitive reserve (estimated through a higher IQ at baseline), and parental education level as protective factors against neuropsychological impairment has been suggested [7, 15, 33, 70, 75]. Over the last decades, emerging research focused on MRI correlates of neuropsychological deficits [7, 19]. Although data are so far sparse and often incongruent, several MRI studies described abnormalities in specific brain areas (i.e., thalamus, hippocampus, amygdala, and cerebellum) of children with cognitive impairment [7, 19]. In an early study in POMS, Till et al. found an association between cognitive impairment (global IQ, processing speed, and expressive vocabulary) and reduced thalamic volume [25].

Beyond cognitive impairment, psychiatric factors and fatigue may contribute to patients’ low functional outcomes. The high prevalence of depression among children and adolescents with MS has been explained with both a direct pathogenic effect of the disease on brain networks and a psychological reaction to the disease [12, 47, 49]. Also, the influence of the disease modifying treatment has been suggested with inconsistent results [12, 43]. Myelin alterations may play a key role in the development of neuropsychiatric disorders [76]. MRI studies suggested that depressive mood disorder may be associated to brain atrophy and demyelinating lesions in temporal [77], parietal [77], and frontal lobes [77]. On the other hand, fatigue, depression, and anxiety may be the result of psychological adjustment to having a chronic illness [78]. A maladaptive response to the high pressures of dealing with this chronic disease may lead to feelings of helplessness and hopelessness and contribute to an increased sense of social isolation [59].

## Limitations

The reviewed literature has several limitations. First, since diagnosis of MS in pediatric age is rare, most studies include small samples. Second, psychological and neuropsychological tools used for patients’ evaluation are extremely heterogeneous. Although research on cognitive profile of POMS has increased over the time and the cognitive assessment has been recommended, the best assessment tools for pediatric MS are still to be determined. Third, a structured assessment of pre-morbid neuropsychological functioning is generally lacking.

## Conclusions

The present review suggests that cognitive dysfunctions are frequent and debilitating symptoms in children and adolescents with MS. They can be worsened by fatigue and psychiatric symptoms, which are also common. Overall, these manifestations may cause considerable functional impairment on daily activities, academic achievement, and quality of life that may require individualized rehabilitative treatment and extensive psychosocial care.
